# Stomatal response to blue light in crassulacean acid metabolism plants *Kalanchoe pinnata* and *Kalanchoe daigremontiana*

**DOI:** 10.1093/jxb/ery450

**Published:** 2018-12-21

**Authors:** Eiji Gotoh, Kohei Oiwamoto, Shin-ichiro Inoue, Ken-ichiro Shimazaki, Michio Doi

**Affiliations:** 1 Department of Forest Environmental Sciences, Faculty of Agriculture, Kyushu University, Motooka, Nishi-ku, Fukuoka, Japan; 2 Department of Biology, Faculty of Science, Kyushu University, Motooka, Nishi-ku, Fukuoka, Japan; 3 Division of Biological Science, Graduate School of Science, Nagoya University, Furo-cho, Chikusa-ku, Nagoya, Aichi, Japan; 4 Faculty of Arts and Science, Kyushu University, Motooka, Nishi-ku, Fukuoka, Japan

**Keywords:** Blue light, CAM plants, phototropins, plasma membrane H^+^-ATPase, signal transduction, stomatal opening

## Abstract

Blue light (BL) is a fundamental cue for stomatal opening in both C_3_ and C_4_ plants. However, it is unknown whether crassulacean acid metabolism (CAM) plants open their stomata in response to BL. We investigated stomatal BL responses in the obligate CAM plants *Kalanchoe pinnata* and *Kalanchoe daigremontiana* that characteristically open their stomata at night and close them for part of the day, as contrasted with C_3_ and C_4_ plants. Stomata opened in response to weak BL superimposed on background red light in both intact leaves and detached epidermal peels of *K. pinnata* and *K. daigremontiana*. BL-dependent stomatal opening was completely inhibited by tautomycin and vanadate, which repress type 1 protein phosphatase and plasma membrane H^+^-ATPase, respectively. The plasma membrane H^+^-ATPase activator fusicoccin induced stomatal opening in the dark. Both BL and fusicoccin induced phosphorylation of the guard cell plasma membrane H^+^-ATPase in *K. pinnata*. These results indicate that BL-dependent stomatal opening occurs in the obligate CAM plants *K. pinnata* and *K. daigremontiana* independently of photosynthetic CO_2_ assimilation mode.

## Teaser

Blue light-specific stomatal opening occurs in obligate crassulacean acid metabolism plants such as *Kalanchoe pinnata* and *Kalanchoe daigremontiana* that characteristically close stomata for part of the daytime.

## Introduction

Stomata are small pores for gas exchange located mainly on plant leaf and stem surfaces. A pair of guard cells regulates stomatal opening and closing and the exchange of CO_2_ and H_2_O between the leaves and the atmosphere. Stomatal movements are controlled by light, CO_2_, humidity, and endogenous phytohormones (Willmer and Fricker, 1996; Shimazaki et al., 2007; Inoue et al., 2017). Light acts as a key environmental cue to induce stomatal opening under natural conditions, where stomata generally open during the daytime and close at night. Stomatal responses to light have been studied in many seed plants, where the stomatal opening in these plants is regulated by two distinct light-activated signaling pathways: a blue light (BL)- and a photosynthetically active radiation-dependent pathway (Willmer and Fricker, 1996; Shimazaki et al., 2007). Each pathway is detected and distinguished under specific light conditions using a dual-beam protocol (Ogawa et al., 1978; Iino et al., 1985; Shimazaki et al., 2007). Photosynthesis-dependent stomatal opening is induced by various light wavelengths. In contrast, BL-dependent stomatal opening is stimulated by weak BL. Since BL also induces photosynthesis, BL-dependent stomatal opening is generally measured by superimposing weak BL on strong red light (RL) that saturates photosynthesis (Ogawa et al., 1978; Iino et al., 1985; Shimazaki et al., 2007). These irradiation conditions effectively detected BL-dependent stomatal opening in *Adiantum capillus-veneris*, *Vicia faba*, and Arabidopsis (Doi et al., 2004, 2006, 2015). We showed that phototropins function as plant-specific BL photoreceptors in the light-dependent stomatal opening and have studied the signaling mechanisms of the stomatal BL response in Arabidopsis (Kinoshita et al., 2001; Doi et al., 2004; Shimazaki et al., 2007; Inoue *et al.*, 2017).

Vascular plants are classified into C_3_, C_4_, and crassulacean acid metabolism (CAM) according to their mode of photosynthetic CO_2_ assimilation (Buchanan and Wolosiuk, 2010). The stomata of CAM plants typically open at night and close during part of the day as an adaptation to arid conditions, in direct contrast to C_3_ and C_4_ plants. Stomatal closure in CAM plants during the day is linked with an increased concentration of intercellular CO_2_ (*C*_i_) via the production of CO_2_, owing to the decarboxylation of organic acids, rather than the effect of light on the stomatal response (Osmond, 1978; Cockburn et al., 1979; von Caemmerer and Griffiths, 2009). In facultative CAM plants such as *Portulacaria afra* and *Mesembryanthemum crystallinum*, stomatal light responses are plastic and vary with photosynthetic metabolism (Lee and Assmann, 1992; Mawson and Zaugg, 1994; Tallman et al., 1997). In these species, both BL-dependent and photosynthesis-induced stomatal responses occur under C_3_ metabolism. Nevertheless, these were absent when *P. afra* and *M. crystallinum* shifted from C_3_ to CAM. In contrast, obligate CAM plants such as *Kalanchoe pinnata*, *Kalanchoe daigremontiana*, and *Aechmea* ‘Maya’ exhibit typical diel gas exchange cycles when they are well hydrated (Dodd et al., 2002; von Caemmerer and Griffiths, 2009; Ceusters et al., 2014). Diel gas exchange in obligate CAM plants consists of four phases: Phase I (nocturnal fixation of atmospheric CO_2_ into organic acids), Phase II (transient assimilation of CO_2_ at dawn), Phase III (release of CO_2_ internally from organic acids in the daytime), and Phase IV (assimilation of CO_2_ in the late afternoon). In association with diel gas exchange, the stomata of obligate CAM plants have been shown to be nearly closed at Phase III, but open at Phases II and IV, which suggests the existence of a stomatal light response in obligate CAM plants (von Caemmerer and Griffiths, 2009; Ceusters et al., 2014). Despite numerous studies on stomatal movements in CAM plants, it is unknown how their stomata open in response to light (Nishida 1963; Dayanandan and Kaufman, 1975; Osmond, 1978; Cockburn et al., 1979; Jewer et al., 1981; Jewer and Incoll, 1981; Lee and Assmann, 1992; Mawson and Zaugg, 1994; Tallman et al., 1997; von Caemmerer and Griffiths, 2009; Ceusters *et al.*, 2014).

Recently, we reported that vascular plants acquired the ability to perform BL-dependent stomatal opening in an early evolutional stage of land plants, more than 400 million years ago (Doi et al., 2006, 2015; Doi and Shimazaki, 2008). It is well known that the stomatal response to BL occurs in both C_3_ and C_4_ plants (Supplementary Fig. S1 at *JXB* online; Ogawa et al., 1978; Grantz and Assmann, 1991; Shimazaki et al., 2007). While BL-dependent stomatal opening does not occur in facultative CAM plants such as *P. afra* and *M. crystallinum* when performing CAM photosynthesis (Lee and Assmann, 1992; Tallman et al., 1997; Ceusters et al., 2014), it remains unclear whether obligate CAM plants have the capacity to open stomata in response to BL. As photosynthesis is a prerequisite for BL-dependent stomatal opening in C_3_ and C_4_ plants, it is interesting to investigate stomatal responses to BL in obligate CAM plants whose photosynthetic mechanisms differ from those of C_3_ and C_4_ plants (Willmer and Fricker, 1996; Shimazaki et al., 2007; Suetsugu *et al.*, 2014).

Here, we investigated the stomatal BL responses of the obligate CAM plants *K. pinnata* and *K. daigremontiana*. With regard to their stomatal responses, it was previously stated that both species show significant daytime stomatal opening in Phases II and IV (von Caemmerer and Griffiths, 2009). We demonstrated that BL-dependent stomatal opening occurs in both species during daylight hours. We also showed that type 1 protein phosphatase and plasma membrane H^+^-ATPase, which participate in the guard cell BL signaling pathways of C_3_ plants like Arabidopsis and *V. faba*, are also crucial for BL-dependent stomatal opening in these CAM plants (Kinoshita and Shimazaki, 1999; Takemiya et al., 2006; Shimazaki *et al.*, 2007).

## Materials and methods

### Plant material and growth conditions

The obligate CAM plants *Kalanchoe pinnata* and *K. daigremontiana* (von Caemmerer and Griffiths, 2009) were grown from epiphyllous buds in plant growth chambers (CLH-301; Tomy Seiko, Tokyo, Japan) under a 12 h light (25 °C)–12 h dark (18 °C) cycle using white fluorescent lamps (100 µmol m^−2^ s^−1^) except as otherwise noted. Experiments were performed on fully expanded leaves from ~3-month-old plants.

### Gas exchange measurements

**Fig. 2.**
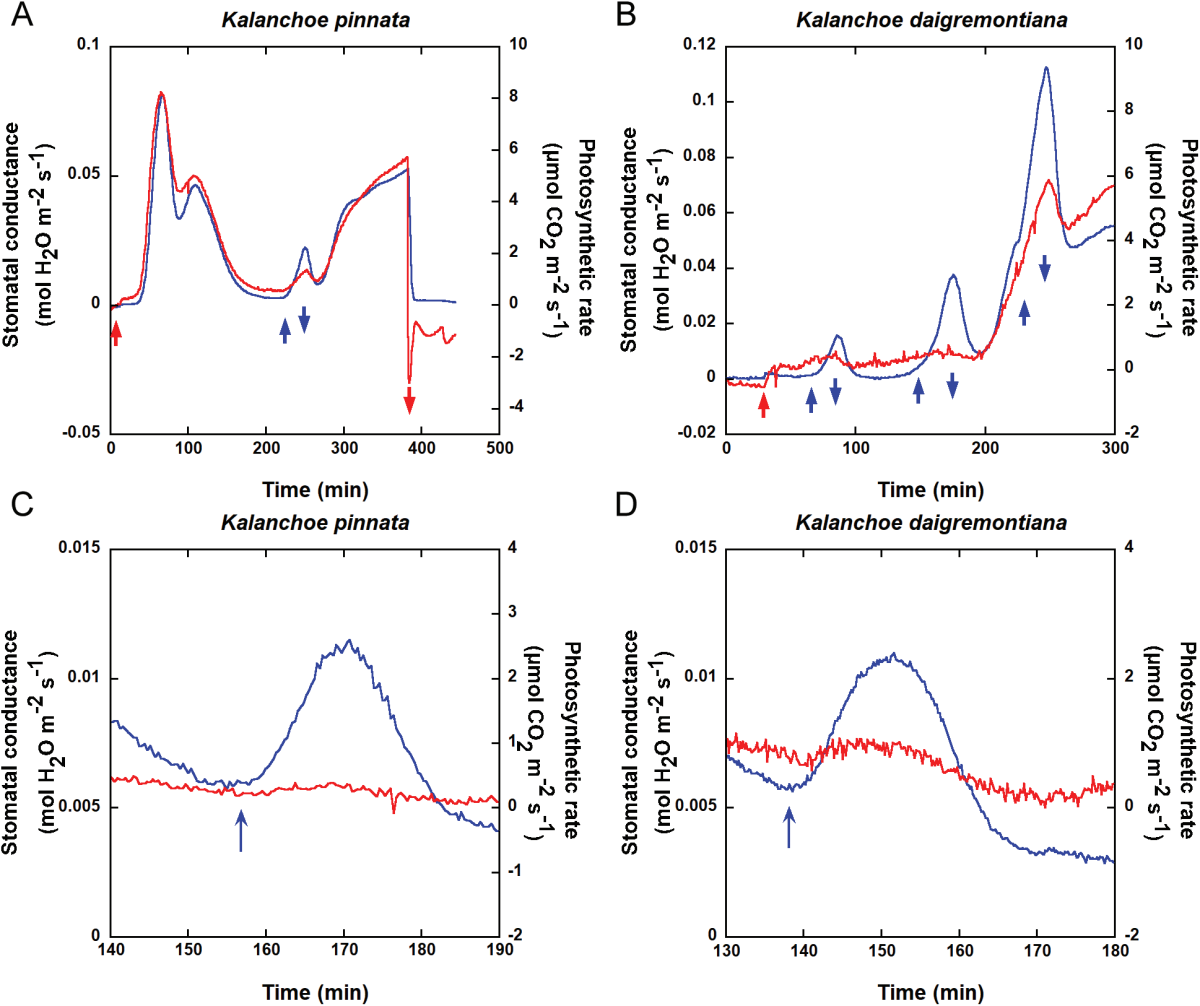
Changes in stomatal conductance (blue line) and photosynthetic rate (red line) in response to light in mature leaves of *Kalanchoe pinnata* (A, C) and *K. daigremontiana* (B, D). *Kalanchoe pinnata* and *K. daigremontiana* were grown from epiphyllous buds in plant growth chambers under a 12 h light (25 °C)–12 h dark (18 °C) cycle with white fluorescent lamps (100 µmol m^−2^ s^−1^). Both plants were maintained in the dark overnight prior to measurements. (A, B) BL at 10 µmol m^−2^ s^−1^ was applied to the upper surface of a leaf (indicated by the upward arrows), and was turned off (indicated by the downward arrows) in the presence of background RL at 600 µmol m^−2^ s^−1^. (C, D) A pulse (100 s) of BL at 150 µmol m^−2^ s^−1^ was applied to the plant leaves in early stage of Phase III at the position of upward arrows in the presence of background RL at 600 µmol m^−2^ s^−1^.

Gas exchange measurements in intact plants were conducted in air-conditioned dark rooms (unless otherwise noted) using an open path gas exchange system (LI-6400; Li-Cor, Lincoln, NE, USA). Plants were maintained in darkness overnight prior to measurements. At the end of Phase I, leaves were clamped into a 2 cm×3 cm gas-tight leaf chamber, and the temperature was maintained at 24 °C. Measurements were conducted under a constant CO_2_ concentration of 365 µl l^−1^, a constant flow rate of 500 µmol s^−1^, and a relative humidity of 50–60% to ensure stable stomatal conductance measurements. A dual-beam protocol was used to distinguish between BL-dependent and photosynthesis-dependent stomatal opening (Ogawa et al., 1978; Iino et al., 1985; Shimazaki *et al.*, 2007). RL (600 µmol m^−2^ s^−1^) and BL (10 µmol m^−2^ s^−1^) were generated by LED and applied to the upper leaf surfaces. RL was irradiated from the end of Phase I until Phase IV. A 15-min BL pulse was superimposed on the RL during Phase II, III, or IV (Fig. 2A, B). A strong 100 s BL pulse (150 µmol m^−2^ s^−1^) was superimposed on the RL early in Phase III (Fig. 2C, D). Photon fluence rates were measured with a LI-2500 light meter fitted with a LI-190SA quantum sensor (Li-Cor). Data were recorded at 20 s intervals and processed with KaleidaGraph v. 4.0 (Synergy Software, Reading, PA, USA). Experiments were run at least three times and representative data are shown.

### Determination of epidermal stomatal apertures

*Kalanchoe pinnata* and *K. daigremontiana* leaves were harvested at the end of Phase I. Epidermis was peeled off mature leaves with forceps under a dim safety light. The peelings were immersed in basal buffer consisting of 10 mM MES, 50 mM KCl, and 0.1 mM CaCl_2_ (pH 6.5) in Petri dishes and maintained in the dark for 2 h. The epidermis was irradiated with RL (600 µmol m^−2^ s^−1^) in either the absence or the presence of BL (10 µmol m^−2^ s^−1^) for 3 h. When the epidermides were treated with tautomycin (2.5 µM) or vanadate (1 mM), they were added to the basal buffer and the reaction proceeded under light irradiation. Fusicoccin (FC; 5 µM) was added to the basal buffer and the epidermis was incubated in darkness for 3 h. Micrographs of over 50 stomata per treatment were taken with a Leica DM500 fitted with a MC120HD camera attachment (Leica, Wetzlar, Germany). The stomatal aperture was defined as the width-to-length ratio of the stomatal pores.

### Light source

White light was generated by fluorescent lamps (FL 40S N-SDL, Panasonic, Osaka, Japan). For gas exchange measurements, stomatal aperture determination, and plasma membrane H^+^-ATPase activation, BL and RL were generated by light-emitting photodiodes ISL-150x150-HBB (λ=470±5 nm) and ISL-150x150-RR (λ=660±5 nm), respectively (CCS, Kyoto, Japan).

### Antibody production

Polyclonal antibodies against *Vicia faba* plasma membrane H^+^-ATPase (Kinoshita and Shimazaki, 1999) and the penultimate phosphorylated Thr947 of Arabidopsis (AHA2) plasma membrane H^+^-ATPase (Hayashi *et al.*, 2011) were raised as previously described.

### Immunohistochemical detection of guard cell plasma membrane H^+^-ATPase

Epidermal peels were obtained from mature *K. pinnata* leaves using forceps under dim safety light. Detached epidermal tissues were immersed in basal buffer in Petri dishes and maintained in the dark for 1 h at 24 °C. Epidermal tissues were irradiated with RL (600 µmol m^−2^ s^−1^) in the absence or presence of BL (10 µmol m^−2^ s^−1^) for 1 h at 24 °C. FC (5 µM) was added to the basal buffer and the epidermal peels were incubated in darkness for 1 h at 24 °C. Immunohistochemical detection of guard cell H^+^-ATPase was then performed as previously described (Hayashi *et al.*, 2011).

### Estimation of fluorescence intensity

Images were obtained using a confocal laser-scanning fluorescence microscope (Digital Eclipse C1; Nikon, Tokyo, Japan) fitted with a CCD camera (DS-Fi1-L2; Nikon) as previously described (Takemiya *et al.*, 2006). The excitation and emission wavelengths for Alexa Fluor 488 were 488 nm and 515–530 nm, respectively. To estimate fluorescence intensities, the same exposure time (2 s) was used for all images. Fluorescence intensities (>30 stomata per treatment) were determined by ImageJ 1.46r (National Institutes of Health, Bethesda, MD, USA; http://imagej.nih.gov/ij/).

### Statistical analyses

All values are expressed as mean ±standard error of the mean (SEM). Comparisons between groups were performed using one-way ANOVA followed by the Tukey–Kramer multiple comparisons *post hoc* test. Comparisons between two groups were performed by Student’s *t*-test. Differences were considered significant at *P*<0.05. Statistical analyses were run in Excel 2011 (Microsoft Corp., Redmond, WA, USA) with the Statcel 3 add-in (Yanai, 2011).

## Results

### Synergistic effect of RL and BL on stomatal movements in Phase III of the diel CAM cycle

**Fig. 1.**
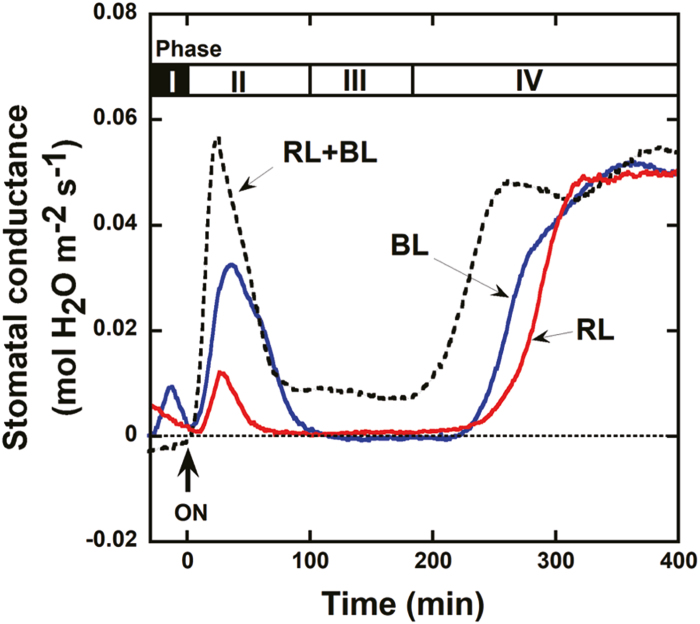
Changes in stomatal conductance in response to light in intact leaves of *Kalanchoe pinnata*. *Kalanchoe pinnata* was cultivated in a greenhouse and maintained in the dark overnight prior to measurements. The arrow pointing up indicates the application of continuous light; red line: 600 µmol m^−2^ s^−1^ red light (RL); blue line: 10 µmol m^−2^ s^−1^ blue light (BL); dotted line: 600 µmol m^−2^ s^−1^ RL and 10 µmol m^−2^ s^−1^ BL (RL+BL). Measurements were replicated a minimum of three times, and representative data are presented.

In the obligate CAM plants *Aechmea* ‘Maya’, *K. pinnata*, and *K. daigremontiana*, the stomata opened at dawn (Phase II) and were fully closed for most of Phase III in response to the dark-to-light transition (von Caemmerer and Griffiths, 2009; Ceusters et al., 2014). We investigated the effect of RL and/or BL on stomatal movements in *K. pinnata* using gas exchange techniques (Fig. 1). When *K. pinnata* was transferred from darkness to continuous RL (600 µmol m^−2^ s^−1^) or BL (10 µmol m^−2^ s^−1^), stomatal conductance transiently increased at Phase II. By Phase III, the stomata were fully closed (Fig. 1; solid lines). The low-fluence rate of RL (10 µmol m^−2^ s^−1^) did not induce stomatal opening in Phases II and III (data not shown; Ceusters *et al.*, 2014). BL (10 µmol m^−2^ s^−1^) combined with strong RL (600 µmol m^−2^ s^−1^) caused a major transient stomatal opening event at Phase II, which prevented full stomatal closure in Phase III (Fig. 1; dotted line). In contrast, full stomatal closure in Phase III was observed in response to either RL or BL irradiation alone. In the presence of both RL and BL, the stomata remained partially open during Phase III until stomata began to re-open widely at Phase IV. The synergy of RL and BL on Phase III stomatal opening suggests that BL-dependent stomatal opening occurs during the daytime in *K. pinnata*.

### BL-dependent stomatal opening in intact CAM plant leaves

Since the stomata of intact *K. pinnata* plants responded to BL, we used a gas exchange technique to investigate stomatal opening in response to BL in *K. pinnata* and *K. daigremontiana.* The comparatively less succulent *K. pinnata* shows greater Phase II and Phase IV stomatal opening than the relatively more succulent *K. daigremontiana* (von Caemmerer and Griffiths, 2009). When stomatal conductance stabilized following *K. pinnata* and *K. daigremontiana* leaf exposure to strong RL, weak BL was superimposed onto the RL according to the dual beam protocol (Fig. 2A, B; Supplementary Fig. S2) to detect any BL-dependent stomatal opening as observed in C_3_ and C_4_ plants (Supplementary Fig. S1). Superimposing weak BL onto strong RL promoted stomatal opening in *K. pinnata* at the end of Phase III, and in *K. daigremontiana* in Phases II, III, and IV. No stomatal conductance changes were observed when RL at the same fluence rate was substituted for BL in the presence of background RL (data not shown). In *K. daigremontiana*, the opening response to BL was greater in Phases III and IV than Phase II. A short BL pulse applied under the RL background caused transient stomatal opening in *K. pinnata* and *K. daigremontiana* (Fig. 2C, D; Supplementary Fig. S3). A pulse of RL applied at the same fluence rate as BL under the RL background did not change conductance (data not shown). Therefore, BL-dependent stomatal opening may occur in intact *K. pinnata* and *K. daigremontiana* leaves.

### BL and fusicoccin induce stomatal opening in CAM plant epidermis

**Fig. 3.**
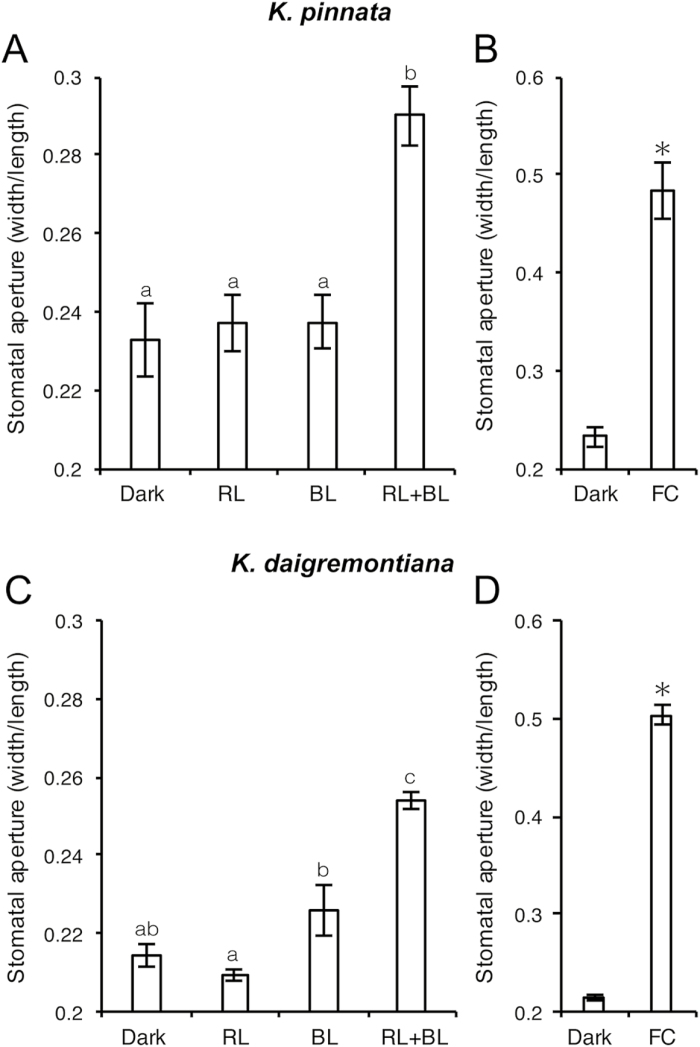
Stomatal responses to light and FC in the epidermis of *Kalanchoe pinnata* (A, B) and *K*. *daigremontiana* (C, D). (A, C) The epidermis was incubated in the dark (Dark), under RL at 600 µmol m^−2^ s^−1^ (RL), under BL at 10 µmol m^−2^ s^−1^ (BL), or both RL and BL at 600 and 10 µmol m^−2^ s^−1^ (RL+BL) for 3 h. (B, D) The epidermis was incubated with 5 µM FC in the dark for 3 h (FC). The sizes of the stomatal apertures of 50–60 stomata were determined microscopically. Data show the mean ±SEM of five independent experiments. Different letters indicate significant differences (*P*<0.05; Tukey–Kramer *post hoc* test).

To determine the BL-dependent stomatal opening mechanism in CAM plants, we explored the effect of light on stomatal movements in epidermal tissues prepared from mature *K. pinnata* and *K. daigremontiana* leaves. One RL (600 µmol m^−2^ s^−1^) or BL (10 µmol m^−2^ s^−1^) application did not induce stomatal opening in isolated epidermal peels. Nevertheless, the combination of BL and RL substantially opened the stomata in *K. pinnata* and *K. daigremontiana* epidermal peels (Fig. 3A, C). Next we tested the effect of the plasma membrane H^+^-ATPase activator fusicoccin (FC) on stomatal movement in the epidermis. FC widely opened stomata and the stomatal opening was more than two times greater than that on exposure to both RL and BL (Fig. 3B, D). These results indicate the occurrence of BL-dependent stomatal opening in the detached epidermises of *K. pinnata* and *K. daigremontiana*, in agreement with the responses observed in intact plants (Fig. 2; Supplementary Figs S2, S3). Furthermore, plasma membrane H^+^-ATPase was suggested to be responsible for BL-dependent stomatal opening in the CAM plants, which is the prevalent mechanism in C_3_ plants.

### Inhibition of BL-dependent stomatal opening by tautomycin and vanadate

**Fig. 4.**
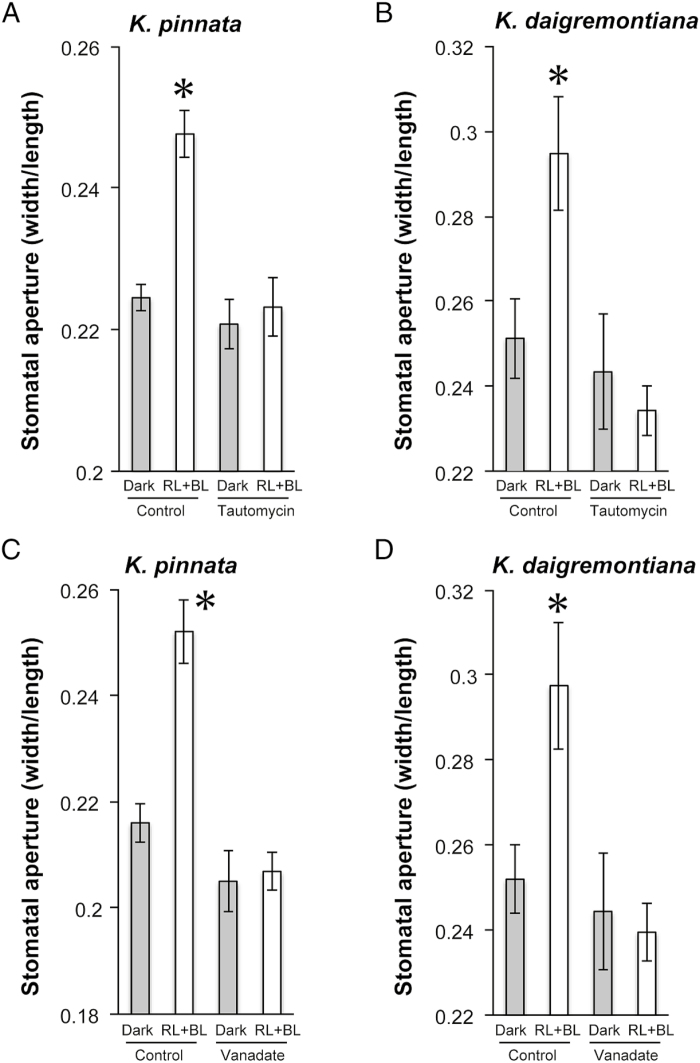
Inhibition of stomatal opening by tautomycin (A, B) and vanadate (C, D) in the epidermis of *Kalanchoe pinnata* (A, C) and *K*. *daigremontiana* (B, D). Epidermal peels were incubated in the dark (Dark), and with both RL and BL at 600 and 10 µmol m^−2^ s^−1^, respectively (RL+BL) for 3 h. Tautomycin and sodium vanadate were added at the final concentration of 2.5 µM and 1 mM, respectively. The sizes of the stomatal apertures of 50–60 stomata were determined microscopically. Data show the means ±SEM of five independent experiments. Asterisks indicate significant differences in comparison with the corresponding dark (control) treatment (*P<*0.05; Student’s *t*-test).

BL-dependent stomatal opening occurs in obligate CAM plants like *K. pinnata* and *K. daigremontiana*. Therefore, the same signaling processes as those observed in Arabidopsis may also be present in these species. The BL signaling pathway includes positive stomatal opening regulators like type 1 protein phosphatase (PP1) and plasma membrane H^+^-ATPase. For this reason, we tested the participation of these enzymes using their inhibitors tautomycin and vanadate, respectively (Bowman and Slayman, 1979; Schwartz et al., 1991; Takemiya et al., 2006). As expected, BL-dependent stomatal opening in the detached epidermis was completely abolished by tautomycin and vanadate in *K. pinnata* and *K. daigremontiana* (Fig. 4). Consequently, PP1 and plasma membrane H^+^-ATPase may function in signaling for BL-dependent stomatal opening in these obligate CAM species as they do in C_3_ plants (Kinoshita and Shimazaki, 1999; Takemiya *et al.*, 2006).

### H^+^-ATPase activation by the BL-dependent signaling cascade in guard cells

**Fig. 5.**
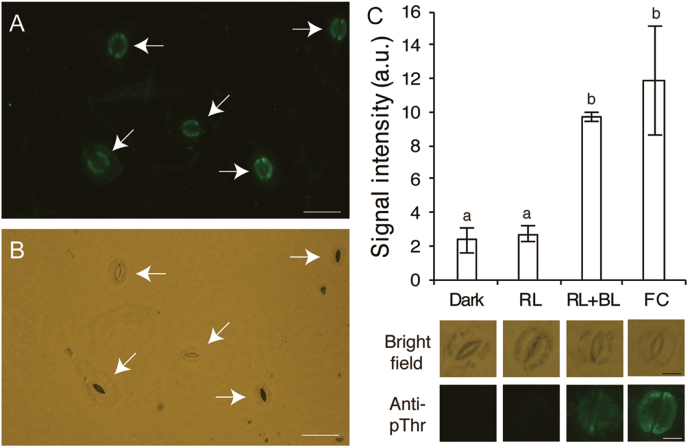
Immunohistochemical detection of phosphorylated H^+^-ATPase in guard cells in the epidermis of *K. pinnata*. (A) Fluorescence image of Alexa Fluor 488 using a polyclonal anti-H^+^-ATPase antibody; (B) bright field of the corresponding epidermis. Scale bars=20 µm. (C) Typical fluorescence images of stomata in the epidermis using an anti-pThr antibody; bright fields of the corresponding stomata in the epidermis (lower) and the quantification of fluorescence intensities of stomata (upper) are shown. Bars indicate means ±SEM of three independent experiments, in arbitrary units (a.u.). The fluorescence intensities of 45 stomata were determined for each condition. Different letters indicate significant differences (*P*<0.05; Tukey–Kramer *post hoc* test). Scale bars=10 µm.

As previously mentioned, the results of the present study show that stomata in the CAM plants *K. pinnata* and *K. daigremontiana* opened in a BL-dependent manner, and that stomatal opening was inhibited by vanadate, an inhibitor of H^+^-ATPase. Since BL is known to activate plasma membrane H^+^-ATPase in guard cells via the phosphorylation of the penultimate threonine in *V. faba* and Arabidopsis (Kinoshita and Shimazaki, 1999; Inoue et al., 2017), we examined the phosphorylation of plasma membrane H^+^-ATPase after treating the epidermis with BL and FC in *K. pinnata*. We subsequently performed immunohistochemical analyses of plasma membrane H^+^-ATPase in the detached epidermis of *K. pinnata* using a polyclonal antibody against plasma membrane H^+^-ATPase from *V. faba* (Hayashi *et al.*, 2011), and showed the exclusive presence of H^+^-ATPase in guard cells (Fig. 5A, B). We then observed the presence of phosphorylated penultimate threonine in the H^+^-ATPase using an antibody against a synthetic H^+^-ATPase peptide with a phosphorylated penultimate threonine via immunohistochemistry (Fig. 5C). Notably, phosphorylated H^+^-ATPase was not detected when the epidermis was maintained under either darkness or RL (600 µmol m^−2^ s^−1^). By contrast, fluorescence signals indicated that the phosphorylation of plasma membrane H^+^-ATPase appeared in guard cells when the epidermis was irradiated with BL (10 µmol m^−2^ s^−1^) in the presence of RL, or incubated with FC in the dark. These results indicate that plasma membrane H^+^-ATPase is present and activated by BL signaling and FC in the guard cells of *K. pinnata*. Although we carefully performed the immunohistochemical experiments using *K. daigremontiana* epidermis, any signal indicating either the presence or the activation of plasma membrane H^+^-ATPase was not obtained at present. The impermeability of the guard cells to antibodies and/or the intercellular environment of ions might have a negative influence on the immunohistochemical detections in *K. daigremontiana*.

## Discussion

A typical diel gas exchange cycle (Phases I to IV) was maintained when the obligate CAM species *Aechmea* ‘Maya’ was transferred from darkness to continuous white light (100 µmol m^−2^ s^−1^) or monochromatic BL (10 µmol m^−2^ s^−1^) (Ceusters et al., 2014). Here, we investigated the stomatal BL responses of the obligate CAM plants *K. pinnata* and *K. daigremontiana* grown under well-hydrated conditions. We found that the stomata of *K. pinnata* were partially open under a combination of RL and BL in Phase III of the diel CAM cycle. In contrast, its stomata entirely closed in response to either RL or BL (Fig. 1). The synergistic effect of RL and BL is characteristic of BL-dependent stomatal opening in C_3_ (Arabidopsis and *Celosia argentea*) and C_4_ (*Zea mays* and *Amaranthus cruentus*) plants (Supplementary Fig. S1; Ogawa et al., 1978; Grantz and Assmann, 1991; Shimazaki *et al.*, 2007). We confirmed that BL-dependent stomatal opening occurs in *K. pinnata* and *K. daigremontiana* using a dual-beam protocol in intact leaves and detached epidermis. Weak BL clearly induced stomatal opening under a background of strong RL (Figs 2, 3; Supplementary Figs S2, S3), indicating the occurrence of BL-dependent stomatal movement in these CAM plants like C_3_ and C_4_ plants. These results suggest that stomatal BL response is conserved in seed plants irrespective of difference in the mode of photosynthetic CO_2_ assimilation.

CAM plants are known to have a high intercellular CO_2_ concentration (*C*_i_) in Phases II and III as a result of the decarboxylation of organic acids, and their *C*_i_ is several orders of magnitude greater than that of ambient air (Cockburn et al., 1979; Spalding et al., 1979). Since most plants generally close their stomata at such high *C*_i_ levels, it is surprising that weak BL was sufficient to induce stomatal opening in these CAM plants, and that this response was greater than the effect of strong RL. Relative differences in diurnal stomatal response to BL should correlate with certain CAM properties like internal organic acid turnover and *C*_i_ levels. Internal organic acid levels reach their maxima by the end of the night (Phase I) and decline toward the end of the day (Phase IV). On the other hand, *C*_i_ is higher in Phase III than it is in Phases II and IV (Cockburn et al., 1979; Spalding *et al.*, 1979). In the present study, the magnitudes of the responses of the stomata in intact *K. daigremontiana* leaves to BL varied with diel CAM cycle phase. They were weakest at the start of Phase III and strongest during Phase IV (Fig. 2). This finding is consistent with the inverse relationship of *C*_i_ with stomatal aperture. *C*_i_, then, may determine the magnitude of diurnal BL-dependent stomatal opening. Since a role of the circadian clock also contributes to the daytime stomatal behavior as well as *C*_i_ (von Caemmerer and Griffiths, 2009), the circadian clock may have a role in the regulation of stomatal BL sensitivity in the CAM plants. In addition, as stomata were already open in phase IV, stomatal responses to BL could be more sensitive than they are in Phases II and III.

Daytime BL-dependent stomatal opening, especially in Phase IV, increased photosynthetic CO_2_ assimilation in CAM plants (Fig. 2A, B, red lines). In the daytime, when evaporative demands are high, stomatal closure prevents excess water loss and therefore enhances water-use efficiency in CAM plants (Osmond, 1978; Borland *et al.*, 2014). At the same time, the reduction of the stomatal aperture also decreases the uptake of water and nutrients required for the growth of plants, and decreasing transpiration causes an increase in leaf temperature. But, on some levels, transpiration dependent on BL-specific stomatal opening in daytime promotes the uptake of minerals and causes leaf cooling.

In the present study, the stomata opened in response to BL in both detached epidermis and intact leaves of *K. pinnata* and *K. daigremontiana* (Figs 2, 3; Supplementary Figs S2, S3). Stomatal BL response was completely inhibited by both tautomycin and vanadate (Fig. 4), which suggests the involvement of PP1 and plasma membrane H^+^-ATPase, respectively, in the signaling cascade of the stomatal response. Immunohistochemistry showed that plasma membrane H^+^-ATPase was localized in the epidermal guard cells (Fig. 5). Moreover, the activation of H^+^-ATPase was also verified by the immunohistochemical detection of phosphorylated penultimate threonine of H^+^-ATPase in response to BL and FC exposures. These results strongly suggest that the obligate CAM plants open their stomata via the same signal transduction cascades that govern BL-dependent stomatal opening in Arabidopsis and *V. faba*. FC also promoted stomatal opening, in addition to the phosphorylation of H^+^-ATPase, which is consistent with the involvement of H^+^-ATPase in BL-dependent stomatal opening.

In conclusion, the obligate CAM plants *K. pinnata* and *K. daigremontiana* opened their stomata in response to BL. Furthermore, PP1 and H^+^-ATPase are involved in the stomatal opening signal transduction cascade in these species. *Kalanchoe pinnata* and *K. daigremontiana* open their stomata in response to BL via a signal transduction cascade analogous to those that occur in C_3_ and C_4_ plants. Therefore, this process is independent of the mode of photosynthesis. Studies of key stomatal regulators in guard cells, such as phototropins, K^+^ channels, and anion channels, may elucidate the mechanisms and physiological significances of BL-dependent stomatal opening in CAM plants.

## Supplementary data

Supplementary data are available at *JXB* online.

Fig. S1. Changes in stomatal conductance and photosynthetic rate in response to blue light in C3 plants (Arabidopsis and *Celosia argentea*) and C4 plants (*Zea mays* and *Amaranthus cruentus*).

Fig. S2. Repeatability of measurement of stomatal conductance and photosynthetic rate corresponding to Fig. 2A, B.

Fig. S3. Repeatability of measurement of stomatal conductance and photosynthetic rate corresponding to Fig. 2C, D.

Supplementary Figures S1-S3

Supplementary Material

## Abbreviations:

BL: blue light
CAM: crassulacean acid metabolism
*C*_i_: intercellular CO_2_ concentration
FC: fusicoccin
PP1: protein phosphatase 1
RL: red light.
