# Supplementary material for: Stomatal response to blue light in crassulacean acid metabolism plants Kalanchoe pinnata and Kalanchoe daigremontiana
Source: J Exp Bot. 2018 Dec 21;70(4):1367–74. doi: 10.1093/jxb/ery450 (PMC6382328; doi:10.1093/jxb/ery450)
Supplement: Supplementary Figures S1-S3 [file ery450_suppl_supplementary_figures_s1-s3.pdf]

Fig. S1

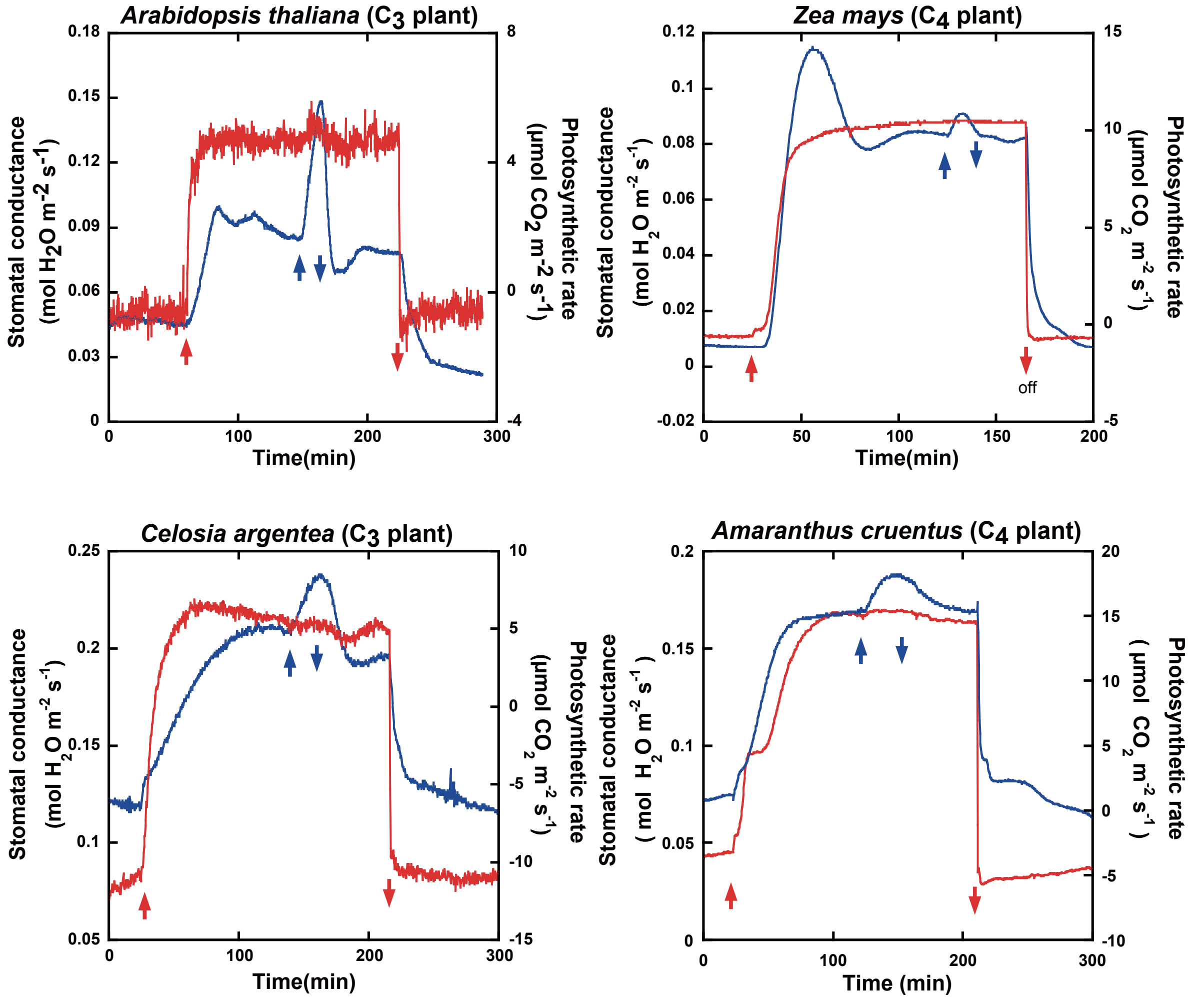

Fig. S1. Changes in stomatal conductance (blue line) and photosynthetic rate (red line) in response to blue light in C3 plants (*Arabidopsis thaliana* and *Celosia argentea*) and C4 plants (*Zea mays* and *Amaranthus cruentus*).

Arabidopsis thaliana (Col-0 ecotype) were grown under a 14 h light:10 h dark cycle at 20–25 °C using white fluorescent lamps (50 µmol m^{-2} s^{-1}) for 4 weeks in a temperature-controlled growth room. *Celosia argentea*, *Amaranthus cruentus*, and *Zea mays* were raised in a plant growth chamber (CLH-301; Tomy Seiko, Tokyo, Japan) under a 12 h light (25 °C):12 h dark (18 °C) cycle using white fluorescent lamps (200 µmol m^{-2} s^{-1}).

BL at 10 µmol m^{-2} s^{-1} was applied to the upper surface of a leaf as indicated by the upward arrows, and turned off as indicated by the downward arrows, under background RL at 600 µmol m^{-2} s^{-1} .

Fig. S2

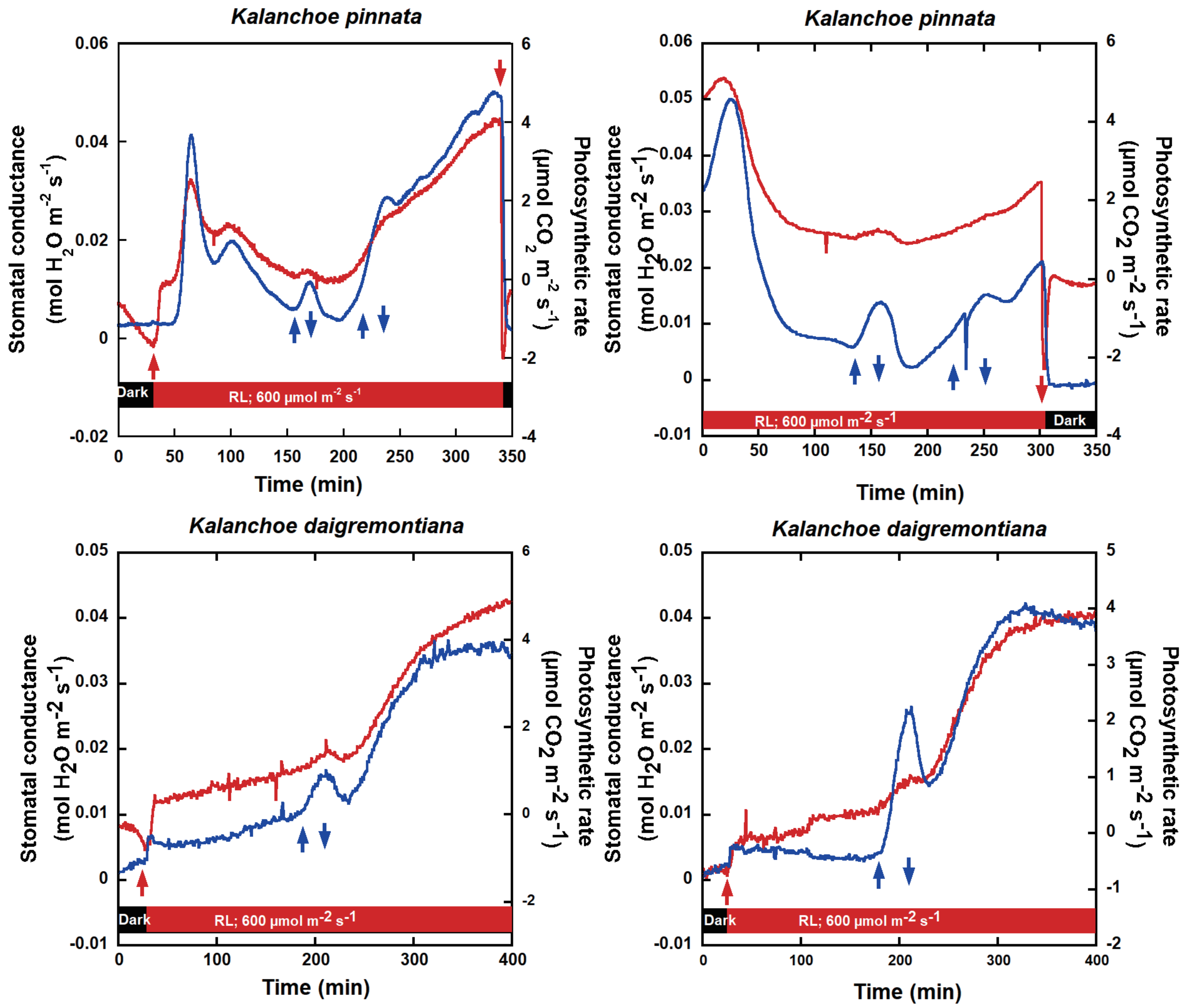

Fig. S2. Repeatability of measurement of stomatal conductance (blue line) and photosynthetic rate (red line) corresponding to Fig. 2 (A and B).
Both *Kalanchoe pinnata* and *K. daigremontiana* were maintained in the dark overnight prior to measurements. BL at 10 μmol m^{-2} s^{-1} was applied to the upper surface of a leaf as indicated by the upward arrows, and was turned off as indicated by the downward arrows in the presence of background RL at 600 μmol m^{-2} s^{-1} .

Fig. S3

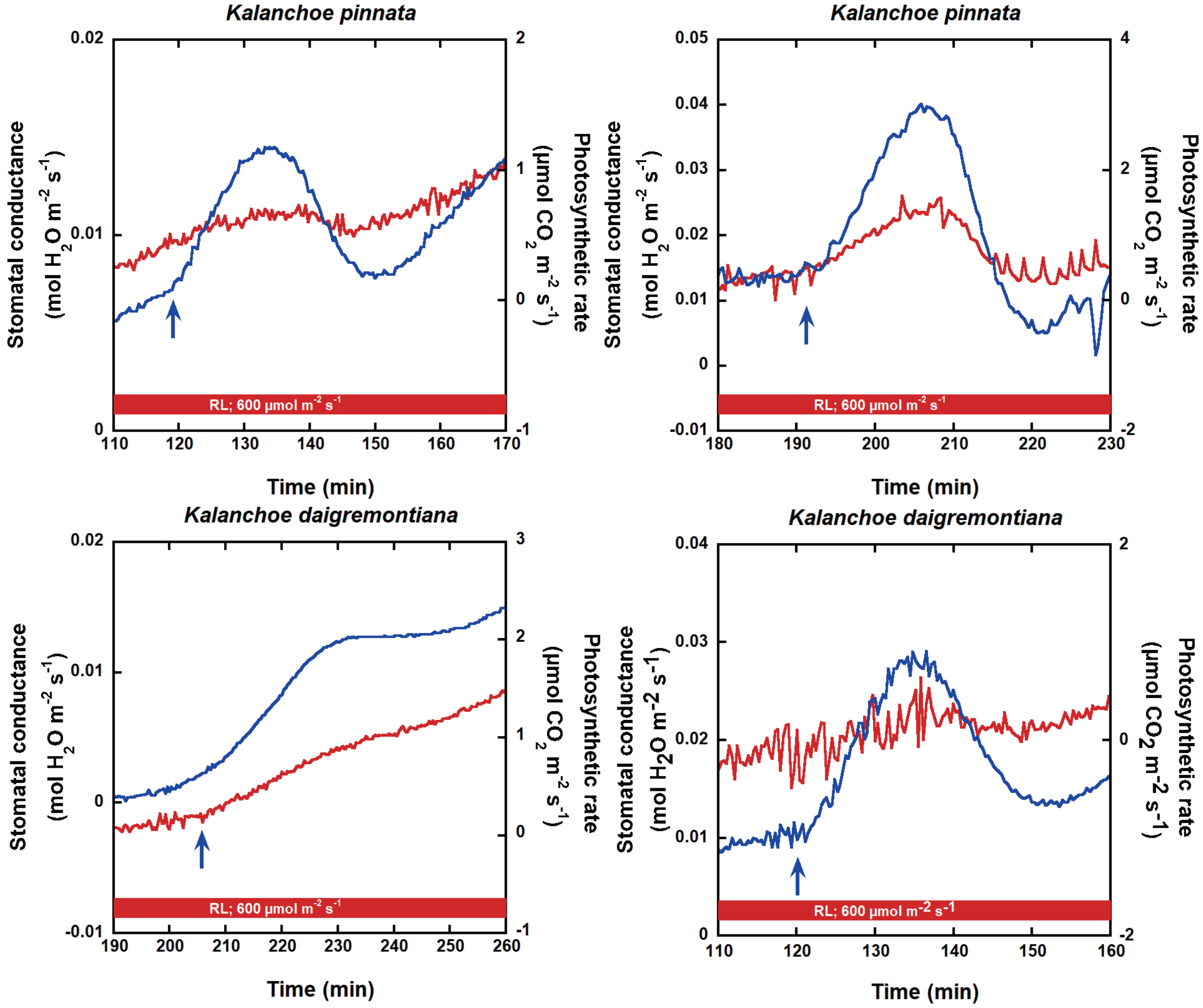

Fig. S3. Repeatability of measurement of stomatal conductance (blue line) and photosynthetic rate (red line) corresponding to Fig. 2 (C and D).
Both *Kalanchoe pinnata* and *K. daigremontiana* were maintained in the dark overnight prior to measurements. A pulse (100 sec) of BL at 150 μmol m^{-2} s^{-1} was applied to the plant leaves in early stage of Phase III at the position of upward arrows in the presence of background RL at 600 μmol m^{-2} s^{-1}.
